# Commentary: The Clinical Impact of the C_0_/D Ratio and the CYP3A5 Genotype on Outcome in Tacrolimus Treated Kidney Transplant Recipients

**DOI:** 10.3389/fphar.2021.603345

**Published:** 2021-02-23

**Authors:** Gerold Thölking, Stefan Reuter

**Affiliations:** ^1^ Department of Internal Medicine and Nephrology, University Hospital of Münster Marienhospital Steinfurt, Steinfurt, Germany; ^2^ Department of Medicine D, Division of General Internal Medicine, Nephrology and Rheumatology, University Hospital of Münster, Münster, Germany

**Keywords:** kidney, metabolism, C/D ratio, tacrolimus, transplantation, renal

## Introduction

Van Gelder et al. nicely reviewed about the influence of the C_0_/D ratio and the CYP3A5 genotype on the outcome of kidney transplant (KTx) recipients treated with tacrolimus (Tac) (van Gelder et al., 2020). The authors report that our group has shown that fast Tac metabolism (C_0_/D ratio <1.05 ng/ml/mg) is associated with a faster decline in renal function, a higher incidence of calcineurin inhibitor nephrotoxicity (CNIT) and BK nephropathy and a lower survival rate (Tholking et al., 2014; Tholking et al., 2016; Schutte-Nutgen et al., 2019; Tholking et al., 2019). They comment that there is no evidence that switching from Tac to a mammalian target of rapamycin inhibitor (mTORi) or other substances improves long-term outcome.

## Conversion of Fast Metabolizers to mTORi

Recently, we published data of 34 KTx recipients (17 patients with a Tac C_0_/D ratio <1.05 ng/mL/mg vs. 17 patients with a C_0_/D ratio ≥1.05 ng/ml/mg (slow metabolizers)) whose therapy was converted to the mTORi everolimus within 24 months after KTx (Tholking et al., 2020). 36 months after switching from immediate-release Tac (IR-Tac) to everolimus, the estimated glomerular filtration rate (eGFR) increased noticeably in fast metabolizers and slow metabolizers, respectively, compared to baseline (+11.0 ± 11.7 (*p* = 0.005) and +9.4 ± 15.9 ml/min/1.73 m^2^ (*p* = 0.049). Adverse events did not differ noticeably between both groups. Although our reported single center sample size data has limitations, we believe that a conversion from Tac to everolimus is safe and beneficial for renal function in KTx recipients who developed CNIT in particular.

## Conversion of Fast Metabolizers to LCPT

In a subsequent study on liver transplant recipients (LTR), we analyzed whether the renal function benefits from an increase of the bioavailability of tacrolimus (von Einsiedel et al., 2020). LTR were converted from IR-Tac or once-daily extended-release Tac (ER-Tac)) to MeltDose® Tac (LCPT). In LTR switched to LCPT, the daily Tac dose decreased by 33.3% and the C_0_/D ratio had increased by 50% 12 months after conversion due to the higher bioavailability of LCPT. Patients converted to LCPT showed a significant increase in mean eGFR already 6 months after the switch (67.5 vs. 65.3 ml/min/1.73 m^2^ at baseline; *p* = 0.029), which was even more pronounced 12 months after the conversion (70.9 vs. 65.3 ml/min/1.73 m^2^ at baseline; *p* = 0.001). We concluded that a conversion from IR-Tac to LCPT increased the C_0_/D ratio and improved renal function in LTR possibly by flattening the Tac blood concentration curve and lowering peak levels.

## Conclusion

Despite limited data on long-term outcome in recipients of solid organ transplants after conversion from IR- or ER-Tac to other immunosuppressants, we have already shown it can be safe and useful in transplant patients with Tac-related adverse effects.
